# Intergroup evaluative bias in facial representations of immigrants and citizens in the United States

**DOI:** 10.1371/journal.pone.0306872

**Published:** 2024-07-24

**Authors:** Ryan J. Hutchings, Imani Morgan, Jeffrey W. Sherman, Andrew R. Todd

**Affiliations:** 1 Department of Psychological and Brain Sciences, Indiana University, Bloomington, Indiana, United States of America; 2 Department of Psychology, University of California, Davis, Davis, California, United States of America; Yeditepe University, TÜRKIYE

## Abstract

We used a reverse-correlation image-classification paradigm to visualize facial representations of immigrants and citizens in the United States. Visualizations of immigrants’ faces were judged by independent raters as less trustworthy and less competent and were more likely to be categorized as a non-White race/ethnicity than were visualizations of citizens’ faces. Additionally, image generators’ personal characteristics (e.g., implicit and explicit evaluations of immigrants, nativity status) did not reliably track with independent judges’ ratings of image generators’ representations of immigrants. These findings suggest that anti-immigrant sentiment and racial/ethnic assumptions characterize facial representations of immigrants in the United States, even among people who harbor positivity toward immigrants.

## Introduction

An estimated 272 million people globally—or about 3.5% of the world’s population—voluntarily or involuntarily live somewhere besides their birth nation, with an estimated 50.7 million immigrants residing in the United States (U.S.) [1]. The immigrant population in the U.S. has grown considerably, from an estimated 4.7% of the total population in 1970 to 13.7% in 2019, rivaling estimates from the 1860s–1920s [2]. Despite (or perhaps because of) the growth in immigrant populations, divisiveness over immigration lies at the core of left—right political divides in the U.S. and many other immigrant-receiving nations. Anti-immigrant rhetoric, including portrayals of immigrants as threats to public health and safety [3], are commonplace. This anti-immigrant sentiment has sparked interest in understanding people’s mental representations of immigrants, which is vital because such representations can predict the treatment of immigrants and immigration policy more generally [4].

Here, we investigated whether anti-immigrant sentiment is recapitulated in mental representations of immigrants’ facial appearance. People mentally represent the physical appearance of social groups, including the faces of prototypical group members. Such facial representations can reflect intergroup evaluative biases, trait-based stereotypes, and lay beliefs about inter-related social categories, such as age, gender, and race/ethnicity [5, 6]. Thus, investigating mental representations of immigrants’ and citizens’ facial appearance may offer a novel entry into understanding anti-immigrant sentiment.

### Anti-immigrant sentiment

A large body of research has documented anti-immigrant sentiment in many immigrant-receiving nations [4]. For example, a Swedish housing study found that applicants with Arab-sounding names received fewer callbacks for rental showings than did applicants with native, Swedish-sounding names, even when equating the applicants on other relevant attributes (e.g., employment, income, marital status) [7]. Lab studies have also revealed that immigrants as a general social category are commonly viewed as lacking both competence and warmth [8] and as threatening [9]. Indeed, social psychological explanations for anti-immigrant sentiment frequently invoke the *perceived* threats posed by immigrants, including threats stemming from competition over economic resources (i.e., realistic threats), health and safety concerns, and challenges to the cultural dominance of the majority group (i.e., symbolic threats) [10, 11]. For example, describing an immigrant group as endangering the ingroup’s job security or as culturally dissimilar from the ingroup (vs. a control description) engenders greater negativity toward that immigrant group [12]. Concerns about immigrants harboring diseases (i.e., contamination threats) [13] elicit feelings of disgust that motivate negativity toward immigrants and support for restrictive immigration policies [14]. Majority group members often favor culturally similar over culturally dissimilar immigrants, in part, because they view the former as less threatening to the cultural dominance of the majority group [15].

Negative verbal descriptions of immigrants, including media portrayals of immigrants as disease carriers, also evoke anti-immigrant sentiment [16]. Perceivers construct more racially/ethnically minoritized mental representations of immigrants after reading portrayals of criminal behavior perpetrated by immigrants than after reading portrayals of immigrant achievements [17]. Similarly, threat-inducing images of immigrants (e.g., large groups of undocumented immigrants at the U.S.–Mexico border) are overrepresented in news media [18] and viewing such images can promote anti-immigrant policy preferences [19].

Just as anti-immigrant sentiment and negative portrayals of immigrants shape mental representations of immigrants’ *character* (i.e., what they’re like), so too may these factors shape representations of immigrants’ *facial appearance* (i.e., what they *look* like). Intergroup evaluative biases and trait-based group stereotypes are commonly integrated into facial representations of groups [20]. For example, ingroup positivity is evident in facial representations linking facial trustworthiness to ingroup status [21, 22]. Conversely, categorizing immigrants as outgroup members may lead to their faces being represented as untrustworthy. Facial representations also may reflect trait-based group stereotypes, particularly when group membership is perceptually ambiguous [23]. Accordingly, representations of immigrants’ faces may incorporate group stereotypes about immigrants’ traits (e.g., incompetent, untrustworthy).

Immigrant facial representations also may reflect default assumptions about perceived immigrant demography. For example, facial representations of welfare recipients reflect both trait-based stereotypes and cultural associations that racialize welfare recipients [24]. Similarly, immigrant facial representations may reflect perceivers’ default beliefs about the racial/ethnic background of prototypical immigrants. Perceivers’ assumptions about immigrant demography often diverge from actual statistical estimates and can predict their attitudes toward immigrants [25]. Such assumptions also may depend on perceivers’ cultural and personal experience with immigrant populations, which is strongly shaped by geopolitical forces. Thus, perceivers from different world regions might have distinct assumptions about the attributes of immigrants, which could feed into their facial representations of immigrants.

### Reverse correlation as a window into facial representations

We investigated facial representations of immigrants and U.S. citizens using a reverse-correlation image-classification paradigm [26], a data-driven approach to extracting the features underlying face classifications. This technique enables researchers to estimate perceivers’ subjective depictions of the prototypical face of a member of a social group, such as immigrants [27]. Unlike procedures in which researchers modify specific stimulus features in a top-down, theory-driven fashion, reverse-correlation procedures capitalize on the construal of randomly distorted stimuli to achieve a bottom-up, data-driven estimation.

Reverse-correlation studies commonly use a two-phase design, wherein one set of participants (i.e., image generators) constructs facial images of a target group (phase 1: image generation), and a second set of participants (i.e., image raters), who lack knowledge about the specific group the image generators visualized, evaluates the images on some dimension of interest (phase 2: image assessment). Researchers then use the image raters’ responses to make inferences about the image generators’ facial representations. Using this two-phase design, reverse-correlation research has elucidated the facial representations of a range of social categories, including demographic categories (e.g., gender [26], race/ethnicity [28], age [29]), specific social groups (e.g., police officers [30], atheists [31], illegitimate voters [32]), and even novel minimal groups [21, 33, 34]. This method also has been used to document associations between certain groups (e.g., welfare recipients) and perceived race/ethnicity [24]. Of relevance here is research documenting that facial representations of immigrants who acculturate to mainstream U.S. culture (vs. those who maintain their heritage culture) are more frequently categorized as White [35], which suggests that assumptions about immigrants’ racial/ethnic background may be integrated into facial representations of immigrants.

Different perceivers may construct different facial representations of the same group. For example, Black perceivers’ representations of police officers’ faces are rated by independent judges as more negative than are White perceivers’ representations [30]. The role of perceivers’ attitudes in shaping their facial representations is less clear. Some studies suggest that perceivers’ explicit and implicit evaluations of groups significantly predict differences in their facial representations of these groups [28, 36]. Other studies, by contrast, have failed to find a significant relationship between perceivers’ group evaluations and their facial representations of that group [37, 38]. For example, one study found that image generators’ personal blatant dehumanization of Arab people did not significantly predict the extent to which image raters viewed their representations of Arab people’s faces as dehumanized [39].

We view reverse-correlation tasks as part of a larger class of indirect, performance-based measurement procedures, which includes the Implicit Association Test (IAT) [40], the Affect Misattribution Procedure (AMP) [41], among others. A notable difference between these other indirect measurement procedures and reverse-correlation tasks is that the former arguably assess *character* representations of a target group (e.g., immigrants), whereas the latter assess *appearance* representations of the group. Additionally, whereas these other indirect measurement procedures constrain participants’ responses via predefined response labels (e.g., good vs. bad) established by the researcher [42], reverse-correlation tasks allow image generators to use any criteria they like for classifying the faces to a group. Accordingly, they can illuminate whether image generators’ evaluative biases seep into their classifications of immigrant faces [27]. Importantly, however, other elements of reverse-correlation tasks, such as the underlying base face, may constrain image generators’ possible decision criteria [43]. Furthermore, the theory-driven attributes and traits selected by researchers necessarily constrain what image raters evaluate in the images during the image-assessment phase.

## The current research

Two primary objectives guided this research. First, we tested whether there are evaluative differences in representations of immigrants’ and U.S. citizens’ faces. Although this issue has been explored for numerous other social groups [27], to our knowledge, no studies have tested whether anti-immigrant sentiment is recapitulated in the facial representations of immigrants. We predicted that immigrant facial representations would be judged less favorably—as less trustworthy and less competent—than U.S. citizen representations.

Second, we tested whether there are differences in the apparent race/ethnicity of immigrant and citizen facial representations. Although past work has examined this question when additional information about acculturation is present [35], to our knowledge, no studies have assessed the role of racial/ethnic assumptions in immigrant and citizen facial representations in the absence of such information. Guided by past research on these groups [35, 44], we expected that representations of immigrants’ faces would be more likely to be categorized as a non-White race/ethnicity than would representations of citizens’ faces, whereas representations of citizens’ faces would be more likely to be categorized as White than would representations of immigrants’ faces.

As a final, more exploratory objective, we tested whether any observed evaluative biases in immigrant and citizen facial representations vary based on characteristics of the image generators, focusing specifically on image generators’ birth nation and their implicit and explicit evaluations of immigrants. Given our racially/ethnically diverse sample of participants in the U.S., we explored the possibility that native-born (i.e., those born in the U.S.) and foreign-born participants (i.e., those born outside the U.S.) have different cultural and personal experiences with immigrants that could shape the valence and perceived race/ethnicity of their facial representations of this group. Additionally, we explored the possibility that participants with more negative implicit and explicit evaluations of immigrants would generate correspondingly negative facial representations of immigrants.

As is common in reverse-correlation research [27], our investigation comprised an image-generation experiment and several image-assessment experiments. In the image-generation experiment, participants viewed hundreds of pairs of noise-laden faces. In one condition, they selected which face in each pair looked more like an ‘immigrant’; in the other condition, they selected which face in each pair looked more like a ‘natural-born U.S. citizen’ (i.e., a person who is a U.S. citizen by birth). We chose the latter term because non-citizens can become citizens through naturalization.

We then used participants’ selections to construct classification images that estimate their facial representations of these groups. The image-assessment experiments (described below) tested our predictions about the valence and the apparent race/ethnicity of the images, using measures of explicit and implicit impressions provided by raters who were naïve about the groups the image generators visualized. In addition, we explored image generators’ explicit and implicit evaluations of immigrants and nativity status as possible predictors of the impressions and race/ethnicity categorizations of their immigrant facial representations.

Analysis code, data, and materials for all experiments are available at this URL: https://osf.io/95n8h/?view_only=6826b122f63d4ddd875c5b7c9ba02e69. We report all manipulations, measures, and exclusions.

## Image-generation experiment (Phase 1)

### Materials and method

#### Ethics statement

The Institutional Review Board at the University of California, Davis approved this research and the informed consent process (IRB ID# 1111880). This research was determined to involve “no more than minimal risk.” In this and all other experiments, all participants read a document describing the experimental procedures and provided consent prior to participating, and they read a debriefing document at the end of the experimental session.

#### Participants (Image generators)

Given uncertainty about formal power analysis procedures for the image-generation phase in reverse-correlation paradigms [34, 45], we set a target sample size using a heuristic of 100 participants per condition, or 200 participants in a two-condition between-subjects design. In total, 218 University of California, Davis undergraduates participated for course credit. Data collection began on March 4, 2019, and concluded on April 12, 2019. After excluding data from one participant who failed to complete the entire reverse-correlation task, the final sample comprised 217 participants (see Table 1 for demographics in all experiments).

**Table 1:** Participant demographics in each experiment.

Experiment	Age			Gender (%)			Race/Ethnicity (%)				
	*N*	*M*	*SD*	Women	Men	Nonbinary	A	B	L	W	M
IG	217	20.2	2.4	74.1	25.0	0.9	59.3	2.8	18.1	12.0	3.7
IA 1	327	37.6	11.9	57.2	41.7	1.0	7.9	10.7	4.8	71.7	1.4
IA 2	228	20.2	2.2	81.6	17.5	0.9	53.1	1.3	24.6	14.9	2.2

*Note*. IG = Image-generation, IA = Image-assessment, *N* = the number of participants included in the data analyses. Some participants did not report their age, gender, or race/ethnicity. For race/ethnicity, A = Asian, Asian American, or Pacific Islander; B = Black or African American; L = Latina/o/e/x or Hispanic; W = White or European American; and M = reported another or more than one race/ethnicity.

#### Reverse-correlation task

Participants first completed a two-image forced-choice reverse-correlation image-classification task [27], during which they were randomly assigned to generate either a facial representation of an immigrant or a facial representation of a natural-born U.S. citizen. On each of 400 trials, participants viewed two adjacent images of degraded faces (512 × 512 pixels) and, depending on between-subjects condition, selected which face looked more like an immigrant or which face looked more like a natural-born U.S. citizen (hereafter, citizen). We informed participants that there were “no right or wrong answers” and urged them to use their “gut-level reactions” when making decisions.

The face stimuli were created with the *rcicr* 0.3.4.1 package [46] by superimposing random noise patterns onto a base-face image. The base face was the mean of all the neutral-expression White male faces from the Averaged Karolinska Directed Emotion Face Database [47]. This gray-scaled, highly averaged White male face has been used frequently in prior reverse-correlation work, including in studies on facial representations of non-White racial/ethnic groups [28, 39]. Previous researchers carefully manipulated the low-level properties of this averaged facial image to match the low-level properties of the specific superimposed noise used in this procedure [27]. Without altering the base face in these ways, the superimposed noise will fail to meaningfully distort the image, leading to low quality classification images. The noise patterns were 4,092 superimposed truncated sinusoid patches in all possible combinations of 2 cycles in 6 orientations (0°, 30°, 60°, 90°, 120°, 150°) × 5 spatial frequencies (1, 2, 4, 8, 16 patches per image) × 2 phases (0, π/2), with random contrasts. A unique noise pattern was generated for each trial and placed atop the base face to create one face stimulus. The other face stimulus on each trial was the inverse of the noise pattern atop the same base face. This strategy maximizes the between-face contrast on each trial [48].

#### Single-Category Implicit Association Test (SC-IAT)

We next assessed implicit evaluations of immigrants with a SC-IAT [49]. Participants completed 5 blocks of trials in which they categorized 4 words related to immigrants (*Immigrant*, *Immigration*, *Nonnative*, and *Nonindigenous*) and 20 evaluative words—10 normatively positive words (*Love*, *Fun*, *Health*, *Vacation*, *Gifts*, *Friends*, *Success*, *Honesty*, *Freedom*, and *Peace*) and 10 normative negative words (*Virus*, *Vomit*, *Cancer*, *Rotten*, *Pollution*, *Enemy*, *Failure*, *Poison*, *Abuse*, and *Stress*) taken from prior research on implicit evaluations of social groups [50–52]. In Block 1 (20 trials), participants categorized words as Bad or Good. In Block 2 (20 trials), they categorized Immigrant words and Bad words using one key and Good words using the other key. The first critical block (Block 3: 40 trials) was identical to Block 2. In Block 4 (40 trials), the label positions changed, and participants categorized Immigrant words and Good words using one key and Bad words using the other key. The second critical block (Block 5: 40 trials) was identical to Block 4.

The SC-IAT was scored using the *D* algorithm [40], with higher scores reflecting more negative evaluations of immigrants. Nine participants did not complete the SC-IAT, and we excluded data from 5 participants whose response times (RTs) were <300 ms on >10% of trials. Trials with RTs >10,000 ms were also excluded (0.2% of trials). SC-IAT scores in the immigrant condition (*M* = 0.13, *SD* = 0.33) and the citizen condition (*M* = 0.18, *SD* = 0.35) did not significantly differ, t(199.69) = 1.04, *p* = .300. These SC-IAT scores served as an indicator of participants’ implicit evaluations of immigrants, which we used as a predictor variable in exploratory analyses reported below.

#### Feeling thermometers

Participants then evaluated various social groups, including immigrants, on a scale from 0 (*very cold*) to 99 (*very warm*). Feelings of warmth toward immigrants in the immigrant condition (*M* = 78.31, *SD* = 21.85) and the citizen condition (*M* = 79.53, *SD* = 21.47) did not significantly differ, *t*(212.55) = 0.41, *p* = .680. These feeling thermometer ratings served as an indicator of participants’ explicit evaluations of immigrants, which we used as a predictor variable in exploratory analyses reported below.

#### Demographics

Finally, participants reported their age, gender identity, racial/ethnic identity, birth nation, and native language. We used participants’ self-reported birth nations to determine whether they were born in the U.S. (native-born) or outside the U.S. (foreign-born).

### Image processing

**Fig 1:**
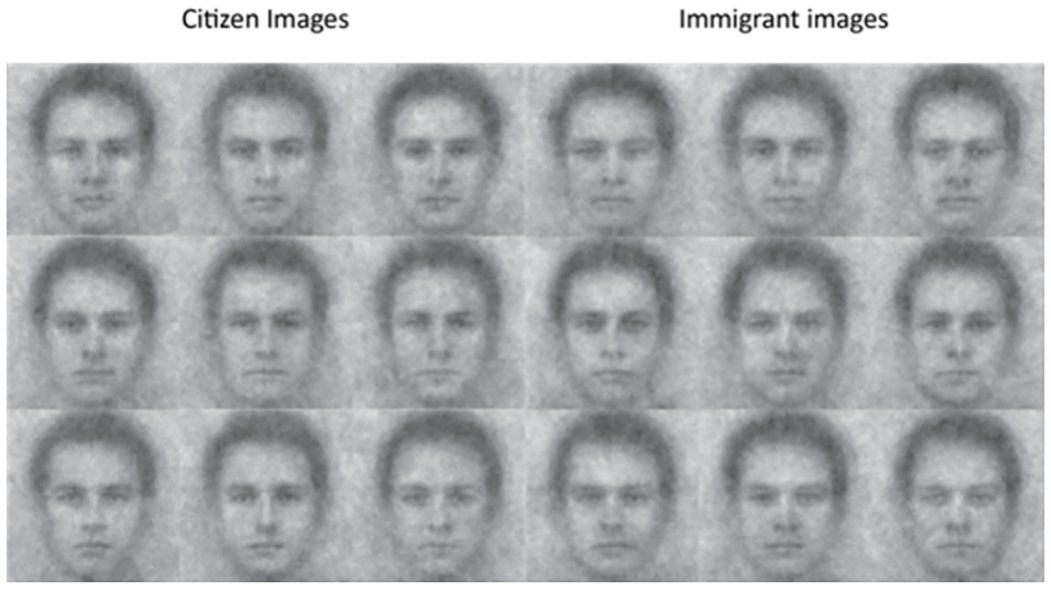
Example individual classification images of citizens and immigrants generated by participants during the reverse-correlation task in the image-generation experiment.

Again using the *rcicr* package [46], we created individual classification images (hereafter, images) that were later evaluated in the image-assessment experiments. We did so by averaging all the selected noise patterns for each participant and superimposing this noise pattern onto the base face. This procedure produced 107 immigrant images and 110 citizen images, or 1 individual image per image generator (for example images, see Fig 1).

Though common in reverse-correlation research, we do *not* report experiments assessing the group images in the citizen and immigrant conditions. Group images can artificially inflate differences between conditions (i.e., between citizen and immigrant conditions) by eliminating all the variance within conditions (i.e., between image generators within the citizen or immigrant conditions), thereby increasing Type-1 error [53].

## Image-assessment experiments (Phase 2)

To test our predictions about differences in facial representations of immigrants and citizens, we conducted two image-assessment experiments with new samples of participants. In the first image-assessment experiment, participants rated the immigrant and citizen individual images on competence, trustworthiness, or perceived race/ethnicity. The second image-assessment experiment aimed to replicate the competence and trustworthiness findings from the first image-assessment experiment, again using the immigrant and citizen individual images but now as primes in a sequential-priming task. In both image-assessment experiments, we explored the relationship between image generators’ explicit and implicit evaluations of immigrants and nativity status and image raters’ impressions of the immigrant images.

## Image-assessment Experiment 1

### Materials and method

#### Participants (Image raters)

In total, 327 Amazon Mechanical Turk (MTurk) workers living in the U.S. participated for modest pay. Data collection began and concluded on April 24, 2019. The final sample, after excluding data from participants who failed to complete the entire image-assessment procedure, comprised 290 participants. Because participants rated images on one of three dimensions (competence, trustworthiness, or perceived race/ethnicity), and because we conducted separate linear mixed-effects models (LMEMs) for each dimension, we used the number of participants in the smallest condition (*n* = 94) to estimate power. A sensitivity power analysis (https://jakewestfall.shinyapps.io/crossedpower/), given a stimuli-within-condition design and default variance partitioning coefficients [54], indicated that 94 participants and 217 images affords 80% power to detect an effect size as small as *d*_z_ = 0.28.

#### Procedure

Participants, who were naïve about what groups the image generators visualized, rated 100 randomly selected individual images on a single randomly assigned dimension: *trustworthiness* (*n* = 97 participants), *competence* (*n* = 94 participants), or *race*/*ethnicity* (*n* = 99 participants). After completing 7 practice trials, participants rated each image (e.g., 1 = *extremely untrustworthy*, 7 = *extremely trustworthy*). For the race/ethnicity condition, participants categorized each image by selecting one of the following labels: African American, Asian, White, Latinx, or Native American.

### Results

#### Primary analyses

Using the *afex* package [55], we fit separate LMEMs for each trait and race/ethnicity categorization (see Table 2); the latter were recoded into separate binary variables for each category label. Each model contained a fixed effect of Immigrant Status (immigrant = -1, citizen = 1). Because the dependent variable was cross-classified—it was nested within both participants (i.e., each participant responded to multiple images) and images (i.e., each image received responses from multiple participants)—the models included by-participant and by-image random intercepts to afford calculation of separate intercepts for each participant and each image. The models also included by-participant random slopes of Immigrant Status to allow the effect of this variable to differ by participants and images. Because image generators classified faces either as immigrants or as citizens, the models did not include a by-image random slope of Immigrant Status. Each LMEM contained the maximal random-effects structure [56] described above, except when noted. Given negligible differences between linear and logistic models [57], we analyzed the race/ethnicity categorization data using linear models for interpretive ease. Coefficients can be interpreted as probabilities.

**Table 2:** Trait impressions and race/ethnicity categorizations by immigrant status in image-assessment Experiment 1.

Rating	Citizen (*SE*)	Immigrant (*SE*)	*B* (*SE*)	*t*	*df*	*p*
Trait Impressions						
Competence	4.49 (0.10)	4.16 (0.10)	-0.163 (0.029)	-5.78	207.03	< .001
Trustworthiness	3.81 (0.09)	3.52 (0.09)	-0.145 (0.029)	-5.05	237.06	< .001
Race/Ethnicity Categorizations						
African American	0.04 (0.01)	0.08 (0.01)	0.021 (0.005)	4.39	175.90	< .001
Asian	0.06 (0.01)	0.09 (0.01)	0.007 (0.004)	1.96	147.90	.052
Latinx	0.16 (0.02)	0.20 (0.02)	0.018 (0.005)	3.30	124.80	.001
Native American	0.05 (0.01)	0.07 (0.01)	0.008 (0.003)	3.18	98.16	.002
White	0.68 (0.03)	0.57 (0.03)	-0.055 (0.010)	-5.47	223.96	< .001

*Note*. *t* values were computed using the Satterthwaite approximation for degrees of freedom implemented in the *afex* package in R.

As displayed in Table 2, immigrant images were rated as both less competent and less trustworthy than were citizen images. In addition, citizen (vs. immigrant) images were more frequently categorized as White, whereas immigrant (vs. citizen) images were more frequently categorized as African American, Latinx, and Native American. Asian categorizations did not significantly differ between immigrant and citizen images.

#### Exploratory analyses

Although our study was powered only with the primary analyses in mind, we also explored whether image generators’ explicit and implicit evaluations of immigrants and nativity status predicted the image raters’ impressions and racial/ethnic categorizations of their immigrant images. To do so, we fit separate LMEMs for each trait (competence and trustworthiness) and race/ethnicity categorization, including ratings of only the immigrant images. The models contained fixed effects of image generators’ explicit immigrant evaluations, image generators’ implicit immigrant evaluations, and image generators’ nativity status (-1 = native-born, +1 = foreign-born). Image generators’ explicit and implicit evaluations of immigrants were mean-centered and scaled by dividing scores by their standard deviations. Such standardization of measures on different scales allows us to compare the relative sizes of the regression coefficients. Again, because the dependent variables were nested within both participants and images, the models included by-participant and by-image random intercepts.

As displayed in Table 3, no significant effects involving image generators’ implicit evaluations of immigrants, explicit evaluations of immigrants, or nativity status emerged on trait impressions of the immigrant images. Similarly, no significant effects involving image generators’ implicit or explicit evaluations of immigrants emerged on the race/ethnicity categorizations of the immigrant images. Furthermore, no significant effects involving image generators’ nativity status emerged on any race/ethnicity categorizations of the immigrant images, with one exception. Immigrant images generated by foreign-born participants (*M* = 0.09, *SE* = 0.01) were slightly more frequently categorized as Asian by image raters than were immigrant images generated by native-born participants (*M* = 0.07, *SE* = 0.01).

**Table 3:** Image generators’ explicit immigrant evaluations, implicit immigrant evaluations, and nativity status predicting image raters’ trait impressions and race/ethnicity categorizations of the immigrant images.

Rating	Predictor	*B* (*SE*)	*t*	*df*	*p*
Competence	Explicit	0.07 (0.04)	1.82	4298	.069
	Implicit	0.01 (0.04)	0.34	4298	.732
	Nativity	-0.03 (0.04)	-0.72	4298	.469
Trustworthiness	Explicit	0.05 (0.04)	1.14	4481	.252
	Implicit	0.01 (0.04)	0.35	4481	.727
	Nativity	-0.01 (0.04)	-0.21	4481	.833
African American	Explicit	<-0.01 (0.01)	-0.37	4560	.708
	Implicit	-0.01 (0.01)	-1.90	4560	.058
	Nativity	<-0.01 (0.01)	-0.34	4560	.733
Asian	Explicit	<-0.01 (0.01)	-0.79	4560	.430
	Implicit	<0.01 (0.01)	0.90	4560	.369
	Nativity	0.01 (0.01)	2.05	4560	.040
Latinx	Explicit	<-0.01 (0.01)	-0.72	4560	.470
	Implicit	<-0.01 (0.01)	-0.54	4560	.589
	Nativity	-0.01 (0.01)	-1.69	4560	.092
Native American	Explicit	-0.01 (<0.01)	-1.26	4560	.208
	Implicit	-0.01 (<0.01)	-1.72	4560	.086
	Nativity	0.01 (<0.01)	1.48	4560	.139
White	Explicit	0.02 (0.01)	1.15	4560	.249
	Implicit	0.02 (0.01)	1.40	4560	.162
	Nativity	<-0.01 (0.01)	-0.23	4560	.815

*Note*. Explicit = Image generators’ explicit evaluations of immigrants (feeling thermometer scores), Implicit = Image generators’ implicit evaluations of immigrants (Single Category Implicit Association Test scores), and Nativity = Nativity status. *t* values were computed using the Satterthwaite approximation for degrees of freedom implemented in the *afex* package in R.

### Discussion

Consistent with anti-immigrant sentiment [4], mental representations of immigrants’ faces were rated as less competent and less trustworthy than were mental representations of citizens’ faces. Immigrant facial representations were also more frequently categorized as Black, Latinx, and Native American, whereas citizen facial representations were more frequently categorized as White.

The extent to which the immigrant images were viewed as competent or trustworthy was largely unrelated to image generators’ evaluations of immigrants or nativity status. The lack of significant relationships between image generators’ characteristics and their representations of immigrants’ faces comports with several other studies that also have found limited evidence for individual differences in facial representations of social groups [37, 39].

Image generators’ nativity status did predict the extent to which immigrant images were categorized as Asian, with immigrant images constructed by foreign-born participants being slightly more likely to be categorized as Asian than immigrant images constructed by native-born participants. This finding might suggest that foreign-born and native-born participants have different assumptions about the prototypical racial/ethnic background of immigrants. Because this effect was small and not hypothesized, however, we refrain from interpreting it pending replication in a confirmatory analysis in future research.

This first image-assessment experiment used direct measures of impressions wherein image raters explicitly rated the competence and trustworthiness of the immigrant and citizen facial images. Considerable evidence indicates that people rapidly form consensual face impressions [58], even when doing so is irrelevant to their focal task goal [59]. Insofar as image raters’ impressions of the immigrant and citizen images are formed spontaneously, evaluative biases should also emerge on indirect measures of such impressions.

## Image-assessment Experiment 2

This next image-assessment experiment examined impressions of competence and trustworthiness spontaneously elicited by the immigrant and citizen facial representations using a variant of the stereotype misperception task (SMT) [60]. The SMT is a sequential-priming task that assesses the biasing effect of semantic/evaluative content (e.g., trait impressions) evoked by prime faces on judgments of unrelated target faces. We tested whether the immigrant images and citizen images, which served as primes in the SMT, evoked differential impressions of competence and trustworthiness.

As in the previous image-assessment experiment, we examined the role of image generators’ explicit and implicit evaluations of immigrants and nativity status on image raters’ impressions of competence and trustworthiness. Image generators’ implicit evaluations of immigrants may better predict image raters’ spontaneous impressions (as measured by the SMT) than image raters’ direct ratings (as in the previous study) of the immigrant images, because spontaneous impressions may be more stimulus-driven and less influenced by any strategic responding to avoid the appearance of bias among the image raters.

### Method

#### Participants (Image raters)

In total, 228 University of California, Davis undergraduates participated for course credit. Data collection began on April 30, 2019, and concluded on May 30, 2019. The final sample, after excluding data from participants who pressed the same key on every trial of the SMT [34], comprised 225 participants. Because we ran separate LMEMs for the competence and trustworthiness conditions, we conducted separate sensitivity power analyses for each condition, each with a stimuli-within-condition design and the default variance partitioning coefficients. Both conditions afforded 80% power to detect an effect size as small as *d*_z_ = 0.27.

#### Procedure and materials

Participants, who were naïve about what groups the image generators visualized, were randomly assigned to complete one of two variants of the SMT [60]—a competence SMT or a trustworthiness SMT—each with the following properties. On each trial, a prime face and target face appeared in rapid succession. The prime faces were the immigrant and citizen individual images from the image-generation experiment, as well as a featureless outline of a human head that is commonly used in this task. For ease of presentation, and following other SMT research [34, 61], we did not include the featureless prime in the analyses reported below. Indeed, it is typical for the featureless prime to elicit negativity [62, 63]. The same was true in our data—the featureless prime evoked less competence (*M* = 0.46, *SD* = 0.50; *t*s > 16.25, *p*s < .001) and less trustworthiness (*M* = 0.48, *SD* = 0.50; *t*s > 2.48, *p*s < .05) than did the citizen or immigrant primes—making it an inappropriate baseline stimulus. The target faces were line drawings of computer-generated facial morphs [64] that were created by morphing 24 unique facial identities with facial features that were +2 *SD* or -2 *SD* from the neutral point in competence or trustworthiness.

**Fig 2:**
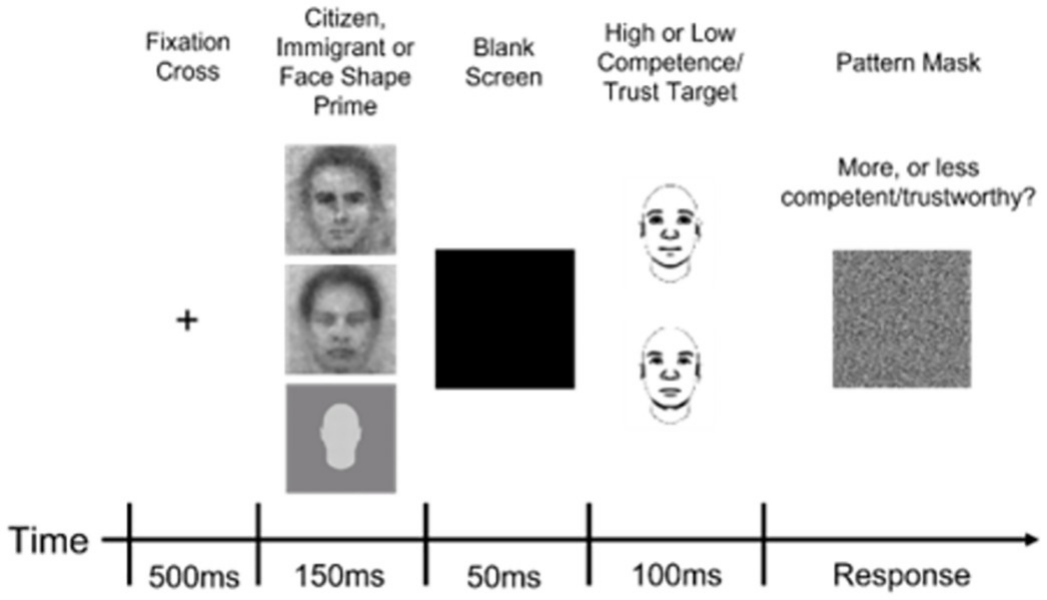
Depiction of the trial sequence in the stereotype misperception task.

After completing two short practice blocks (5 trials and 12 trials, respectively), participants completed two extended testing blocks (132 trials each). Each trial began with a fixation cross (500 ms), followed by a prime face (individual immigrant or citizen image; 150 ms), a blank screen (50 ms), and then a target face (100 ms). Finally, a pattern mask appeared until participants judged the target face as “more competent” or “less competent” (in the competence SMT) or as “more trustworthy” or “less trustworthy” (in the trustworthiness SMT) than the average target face (see Fig 2 for a depiction of the trial sequence).. Each testing block contained 54 trials with immigrant face primes, 54 trials with citizen face primes, and 24 trials with the featureless face prime. Because there were more individual images than SMT trials, a randomly selected individual image appeared on each trial. Trial order was randomized, and immigrant and citizen image primes appeared equally often with target faces of each type.

Task instructions directed participants to remember the prime faces for a later task but to avoid letting these faces affect their judgments of the target faces. Participants were also urged to respond quickly and to use their “gut” feelings when judging the target faces. The proportion of “more competent” judgments (in the competence SMT) or “more trustworthy” judgments (in the trustworthiness SMT) after each prime face served as an indirect index of its competence/trustworthiness. Spontaneous impressions of the prime faces are revealed by their unintended influence on judgments of the target faces [60, 65].

### Results

#### Primary analyses

We fit identical LMEMs for the competence and trustworthiness SMTs. Each model contained a fixed effect of Immigrant Status as well as random intercepts for participants and images and a by-participant random slope for Immigrant Status primes. Because immigrant images and citizen images were generated by separate image generators, we did not fit a by-image random slope of Immigrant Status. Because of convergence issues, we modified the random-effects structure of the trustworthiness SMT by eliminating the correlation between the by-participant random slope of Immigrant Status and the by-participant random intercept.

The immigrant image primes (*M* = 0.59, *SE* = 0.02) spontaneously elicited slightly weaker competence impressions than did the citizen image primes (*M* = 0.60, *SE* = 0.02), *B* = -0.008, *SE* = 0.004, *t*(92.15) = -2.15, *p* = .034. Similarly, the immigrant image primes (*M* = 0.50, *SE* = 0.02) also elicited slightly weaker trustworthiness impressions than did the citizen image primes (*M* = 0.52, *SE* = 0.01), *B* = -0.009, *SE* = 0.004, *t*(123.80) = -2.23, *p* = .028.

#### Exploratory analyses

Next, we explored whether image generators’ explicit and implicit evaluations of immigrants and nativity status predicted image raters’ impressions of their immigrant images. We fit separate LMEMs for the competence and trustworthiness SMTs, including spontaneous impressions of only the immigrant images. The models contained fixed effects of image generators’ explicit evaluations of immigrants, implicit evaluations of immigrants, and nativity status (-1 = native-born, +1 = foreign-born). Again, because the dependent variables were nested within both participants and images, the models included by-participant and by-image random intercepts.

As shown in Table 4, image generators’ explicit evaluations of immigrants significantly predicted image raters’ spontaneous trustworthiness impressions: Image generators with warmer feelings toward immigrants created more trustworthy-looking immigrant images than did image generators with colder feelings towards immigrants. Image generators’ implicit immigrant evaluations and nativity status, by contrast, did not significantly predict image raters’ spontaneous trustworthiness impressions. There were no significant relationships between image generators’ explicit immigrant evaluations, implicit immigrant evaluations, or nativity status and image raters’ spontaneous competence impressions.

**Table 4:** Image generators’ explicit immigrant evaluations, implicit immigrant evaluations, and nativity status predicting image raters’ spontaneous competence and trustworthiness impressions of the immigrant images.

Rating	Predictor	*B* (SE)	*t*	*df*	*p*
Competence	Explicit	<-0.001 (<0.001)	-0.16	11806	.875
	Implicit	-0.004 (0.016)	-0.25	11806	.800
	Nativity	-0.005 (0.006)	-0.82	11806	.414
Trustworthiness	Explicit	0.0005 (0.0002)	2.24	11200	.025
	Implicit	-0.004 (0.013)	-0.33	11200	.743
	Nativity	-0.003 (0.005)	-0.63	11200	.530

### Discussion

These results complement those from the first image-assessment experiment by documenting that evaluative biases in facial representations of immigrants and citizens extend to image raters’ spontaneously elicited impressions of these groups. The immigrant images evoked less competence and less trustworthiness than did the citizen images. Granted, these differences were small, but they were sufficiently apparent to alter image raters’ impressions even after very brief exposures.

Also, as in the first image-assessment experiment, the image-generator characteristics that we examined were not consistently associated with impressions of their immigrant images. These null relationships comport with evidence from several studies that have failed to find significant relationships between image generators’ attitudes and facial representations of social groups [37–39].

## General discussion

The current research used a reverse-correlation image-classification paradigm [26] to investigate facial appearance representations of immigrants and citizens in the United States. We examined (a) whether there were evaluative differences in representations of immigrants’ and citizens’ faces, (b) the perceived race/ethnicity of those immigrant and citizen facial representations, and (c) the potential role of image generators’ implicit and explicit evaluations of immigrants and nativity status in shaping their facial representations of immigrants.

### Summary of findings

In two image-assessment experiments, image raters, who were unaware that the images depicted immigrants and citizens, evaluated the immigrant images less favorably than the citizen images. Specifically, image raters viewed the immigrant images as less competent and less trustworthy than the citizen images, and this pattern of bias emerged on both direct (rating scales) and indirect (sequential priming task) measures of competence and trustworthiness impressions. These findings align with research on negative character representations of immigrant, including stereotypes linking immigrants with low competence and low warmth [8]. Importantly, we used a meaningfully different measure to capture appearance representations of immigrants’ faces. In contrast to previous studies examining character representations (e.g., group stereotypes), participants in our image-generation experiment were not prompted to evaluate the competence or warmth of immigrants, but only to classify images as looking like immigrants (or like U.S. citizens). Yet, negative group stereotypes appear to have guided their classification decisions, suggesting that perceivers may (unknowingly) incorporate these biases into their facial representations.

The immigrant and citizen facial representations also offered insights into assumptions about the racial/ethnic background of prototypical immigrants in the U.S. Image raters more frequently categorized the immigrant images (vs. the citizen images) as a race/ethnicity other than White—as Black, Latinx, or Native American. In contrast, they more frequently categorized the citizen images (vs. the immigrant images) as White. These findings complement prior work linking mental representations of being American and being White [44]. Similarly, the current results mirror previous findings on the role of perceived acculturation on representations of immigrants’ faces [35]. In the absence of information about acculturation, image generators constructed immigrant facial images that were frequently categorized as a race/ethnicity other than White. These results are informative regarding default racial/ethnic assumptions about immigrants in the U.S. Future research should examine the similarity between generic immigrant images and images of more specific immigrant groups [66]; understanding the similarities and differences between these conditions can more clearly illuminate default assumptions about immigrants.

In addition, differences in the perceived race/ethnicity of immigrant and citizen facial representations may have implications for research on American identity denial, the phenomenon whereby Americans from some racial/ethnic groups (e.g., Asian and Latinx Americans) are viewed as less American (or more foreign) than White Americans [67, 68]. It is noteworthy that our image-generator sample included sizable numbers of Asian and Latinx participants, constituting greater than 75% of the sample. Even in this diverse sample, however, citizens were predominantly mentally represented as White. These results seemingly conflict with previous findings that people visually self-project themselves onto their visualizations of superordinate group faces (U.S. citizens) [69]. To our knowledge, however, no research has explored whether facial representations of social groups reflect cultural versus personal representations of those groups. A fruitful avenue for future research could be to explore whether immigrant facial representations differ when image generators are prompted to consider cultural messages (i.e., what U.S. society thinks immigrants look like) versus their own personal beliefs (i.e., what they personally think immigrants look like) [70].

With few exceptions, the characteristics of the image generators (i.e., their implicit and explicit evaluations of immigrants and their nativity status) did not reliably track with the perceived competence, trustworthiness, and race/ethnicity of their immigrant facial representations. The lack of a role of image generators’ implicit evaluations conflicts with some previous findings, including one study indicating that implicit evaluations of Moroccan and Chinese people predict facial representations of those groups [28]. Notably, however, this prior study used procedures to construct aggregated classification images of higher-prejudice and lower-prejudice perceivers that may inflate differences between these two groups [53]. Should individual differences in implicit immigrant evaluations contribute to immigrant facial representations, these contributions may be small, and thus our image-generation experiment might have been underpowered to detect them. It is also possible that they are difficult to observe in individual classification images in part because of the limited reliability of each measurement procedure. A related possibility for the observed null relationships between implicit evaluations and facial representations of immigrants is suggested by research documenting low between-participant variability in performance-based tasks (e.g., Stroop task), despite their ability to produce robust experimental effects [71]. Yet another possibility is poor structural fit [72] between the measure of implicit evaluations (i.e., image generators’ immigrant—good/bad associations) and the measure of facial representations (i.e., image raters’ impressions of the competence/trustworthiness of the immigrant images).

Accordingly, future research that examines the potential role of implicit (or explicit) evaluations in shaping facial representations might benefit from greater structural fit between measures, larger samples of image generators, multiple assessments of implicit evaluations and facial representations [73, 74], an image-processing approach that protects against Type-1 error inflation and strikes a balance between reducing noise and maintaining some image-generator variability (i.e., subgroup images) [45, 53, 75], or other procedures that maximize true-score variance and minimize error variance on these tasks. Such procedures could also be helpful for research aiming to predict downstream implications of biased facial representations (e.g., support for different immigration policies).

### Limitations and future research directions

We acknowledge several additional limitations of this work, each of which suggests potential directions for future research. First, although our image-assessment experiments consistently revealed differences in facial representations of immigrants and citizens, our conclusions rest on a single image-generation experiment—a limitation that characterizes much reverse-correlation research (but see refs. [33, 39, 76] for recent exceptions). Because the results of our image-assessment experiments necessarily depend on the facial representations constructed in the image-generation experiment, future research that replicates the image-generation experiment with a new sample of participants could provide greater clarity on the robustness of our results.

Second, consideration should be given to our sample of image generators, who were recruited from a university participant pool in the Western U.S. and thus are not representative of the U.S. population. Unlike the general U.S. population, our image-generator sample predominantly identified as Asian and women and were mostly young adults. Although we did not assess participants’ political orientation, most students in this research population identify as politically left of center. A different sample of participants might have generated appreciably different facial representations of immigrants and citizen. For example, older participants might bring to mind different facial features when imagining immigrants, based on their age cohort’s experiences with an ever-changing immigrant population. Although most immigrants currently living in the state where the research was conducted (i.e., California) were born in Asia and Latin America [77], immigrant populations differ in other parts of the U.S. and elsewhere around the world. It is possible, for example, that American and European participants mentally represent immigrants differently, given differences in the immigrant populations in those world regions. Some people may also have more negative or positive mental representations of immigrants based on their personal connection to immigration. Perhaps people who strongly self-identify as immigrants would construct positive facial representations of this ingroup. Future research should explore these possibilities.

A related and equally important direction for future research is to explore whether image generators with different racial/ethnic backgrounds construct meaningfully different facial representations of immigrants versus citizens. Although our participant sample was more racially/ethnically diverse than the modal convenience sample of university students, the upshot of this sample diversity is that the absolute number of image generators of some races/ethnicities (e.g., Latina/o/e/x, White, Black) was low, which precludes appropriately powered analyses based on image generator race/ethnicity.

Third, our findings were surely constrained by our use of a White male base face. As noted earlier, this base face has been used extensively in prior research examining facial representations of non-White racial/ethnic groups [28, 39]. Furthermore, its low-level properties were carefully manipulated in prior work to match the low-level properties of the specific superimposed noise used in this procedure, which helps ensure higher quality classification images [28]. Nevertheless, it is possible that using a base face that aggregates (e.g., via morphing) faces of different apparent races/ethnicities [78] would have produced different results. Future research should explore this possibility.

Fourth, an important procedural feature of reverse-correlation research is that participants in the image-assessment phase are naïve about how the facial images were constructed during the image-generation phase. That is, the image raters were unaware that the images they evaluated comprised the image generators’ facial representations of immigrants and U.S. citizens. Accordingly, it remains for future research to determine whether the image raters spontaneously encoded the faces in terms of these social categories, or if they would be able to classify the images in terms of the image generators’ assigned experimental condition (i.e., immigrant vs. citizen) with above-chance accuracy.

Finally, because our interest was in capturing facial representations of the generic category “immigrants,” we focused on how immigrants are mentally represented in the absence of additional information (e.g., country of origin, documentation status). Indeed, it is possible that providing such information about the immigrant group might alter the resulting facial representations [66]. For example, documentation status might affect the positivity of immigrant images, given that prejudice is generally stronger toward undocumented than documented immigrants [79]. Similarly, insofar as some labels for describing immigrants (e.g., “illegal alien”) may connote more negativity than other labels (e.g., “undocumented immigrant”), the latent assumptions in these labels could lead to more negative (positive) immigrant facial representations [80]. Future research that compares facial representations of differently labeled immigrant groups (e.g., documented vs. undocumented) with those of a general, unlabeled immigrant category could provide insights into the diversity of groups that come to mind when imagining immigrants.

## Conclusion

In sum, the current investigation examined facial representations of immigrants and citizens in the United States, finding that these representations recapitulated anti-immigrant sentiment and racial/ethnic assumptions about immigrants. In contrast to some past work, characteristics of the image generators, including their implicit and explicit evaluations of immigrants and nativity status, did not consistently track with image raters’ impressions of the image generators’ immigrant facial representations. That is, image generators with relatively positive feelings toward immigrants did not create meaningfully more positive-looking immigrant facial representations. This work offers initial evidence that anti-immigrant sentiment may seep into mental pictures of immigrants’ faces, even for people who otherwise espouse positive views of immigrants. In doing so, our findings help build toward a broader understanding of the evaluative biases commonly directed toward immigrants.
